# LMW-PTP targeting potentiates the effects of drugs used in chronic lymphocytic leukemia therapy

**DOI:** 10.1186/s12935-019-0786-1

**Published:** 2019-03-21

**Authors:** Nagaja Capitani, Giulia Lori, Paolo Paoli, Laura Patrussi, Arianna Troilo, Cosima T. Baldari, Giovanni Raugei, Mario Milco D’Elios

**Affiliations:** 1 Department of Experimental and Clinical Medicine, University of Florence, Florence, Italy; 2 Department of Experimental and Clinical Biomedical Sciences, University of Florence, Florence, Italy; 3 Department of Life Sciences, University of Siena, Siena, Italy

**Keywords:** Morin, Chronic lymphocytic leukemia, Apoptosis, LMW-PTP, Migration

## Abstract

**Background:**

Low molecular weight protein tyrosine phosphatase (LMW-PTP) is overexpressed in different cancer types and its expression is related to more aggressive disease, reduced survival rate and drug resistance. Morin is a natural polyphenol which negatively modulates, among others, the activity of LMW-PTP, leading to the potentiation of the effects of different antitumoral drugs, representing a potential beneficial treatment against cancer.

**Methods:**

LMW-PTP levels were measured by immunoblot analysis both in CLL cells from patients and in chronic lymphocytic leukemia (CLL)-derived Mec-1 cells. Cell viability was assessed in Mec-1 cells treated with morin alone or in combination with either fludarabine or ibrutinib or following siRNA-mediated LMW-PTP knockdown. Furthermore, the expression levels of VLA-4 and CXCR4 were assessed by both qRT-PCR and flow cytometry and both adhesion to fibronectin-coated plates and migration toward CXCL12 were analyzed in Mec-1 cells treated with morin alone or in combination with fludarabine or ibrutinib.

**Results:**

We observed that LMW-PTP is highly expressed in Mec-1 cells as well as in leukemic B lymphocytes purified from CLL patients compared to normal B lymphocytes. Morin treatment strongly decreased LMW-PTP expression levels in Mec-1 cells and potentiated the anticancer properties of both fludarabine and ibrutinib by increasing their apoptotic effects on leukemic cells. Moreover, morin negatively regulates adhesion and CXCL12-dependent migration of Mec-1 cells by affecting VLA-4 integrin expression and CXCR4 receptor recycling.

**Conclusions:**

Morin treatment in CLL-derived Mec-1 cell line synergizes with conventional anticancer drugs currently used in CLL therapy by affecting leukemic cell viability and trafficking.

**Electronic supplementary material:**

The online version of this article (10.1186/s12935-019-0786-1) contains supplementary material, which is available to authorized users.

## Background

Low molecular weight protein tyrosine phosphatase (LMW-PTP) is an enzyme involved in cell proliferation control by dephosphorylating tyrosine kinase receptors and docking proteins involved in cell adhesion and regulation of gene expression [1]. In the last decades, compelling evidence suggested that LMW-PTP has an important role in modulating cancer cell responses such as apoptosis inhibition, cell motility and glucose metabolism. It is indeed overexpressed in different cancers, including colon cancer and neuroblastoma, and its expression is related to worse prognosis and reduced survival rate [2]. Similar results were obtained in a rat model of colorectal cancer, where strongly increased LMW-PTP expression was already observable in pre-neoplastic lesions [3]. Interestingly, LMW-PTP also contributes to regulation of the glucose metabolism in different human cancer cells [4]. More recent studies demonstrated that LMW-PTP is involved in the regulation of apoptosis and in the acquisition of drug resistance in many cancers [5]. Moreover, high levels of LMW-PTP, usually associated with aggressive cancer, induce resistance to chemotherapy. siRNA-mediated knock-down of LMW-PTP strongly reduces the malignant potential of colon cancer cells [6, 7]. These data strongly suggest that targeting LMW-PTP phosphatase could be of potential interest to amplify the effects of the conventional therapies also in other types of cancer.

Chronic lymphocytic leukemia (CLL) is the most common B-cell neoplasm in Europe and United States, characterized by progressive accumulation of monoclonal CD5+ B cells in peripheral blood, bone marrow, and peripheral lymphoid organs. CLL has a highly variable clinical course, ranging from a stable disease to a progressive, severe and almost invariably fatal disease [8, 9], with subsequent strong implications for drug regimens, which are also highly variable. Together with the conventional drugs such as fludarabine, cyclophosphamide, chlorambucil and others [10], clinical trials have recently identified more specific inhibitors of B cell receptor (BCR) signaling such as ibrutinib, a Bruton tyrosine kinase (Btk) inhibitor [11, 12], idelalisib, a phosphoinositide 3-kinase (PI3K) inhibitor [13], and venetoclax, a Bcl-2 inhibitor [14], as effective alternatives to current chemoimmunotherapy-based regimens Because of the increasing side effects or resistance to drugs used for managing CLL, new natural compounds were recently tested for CLL treatment [15].

Morin, a bioactive flavonoid member of the family of Moraceae, has been reported to be endowed of peculiar pharmacological properties [16]. Compelling evidence demonstrated that morin is a bioactive compound, showing a broad range of pharmacological activities and very low cytotoxicity by modulating the activity of many enzymes. In some cases, morin shows a systemic protective action, reducing negative side effects of several drugs, without interfering with their functions [17]. Moreover, we have recently demonstrated that morin reduces LMW-PTP protein levels and sensitizes melanoma cells to chemo- and radiotherapy [7]. In addition, we have also shown an in vivo action of Morin on colon carcinoma in a Pirc rats model system [18].

In this study we assessed the effect of morin on LMW-PTP expression in the CLL-derived Mec-1 cell line, demonstrating that morin potentiates the pro-apoptotic and anti-migratory effects of fludarabine and ibrutinib, two drugs currently used in CLL treatment.

## Methods

### Cells, antibodies and reagents

CLL-derived B-cell line Mec-1 were cultured in Roswell Park Memorial Institute (RPMI) with 7.5% bovine calf serum (BCS). Peripheral blood samples were collected from 9 patients satisfying standard morphologic and immunophenotypic criteria for CLL. B cells from 10 buffy coats were used as healthy controls. At collection, patients had never received treatment. Primary B cells were purified by negative selection using RosetteSep B-cell enrichment Cocktail (StemCell Technologies, Vancouver, Canada) followed by density gradient centrifugation on Lympholite (Cedarlane Laboratories, The Netherlands).

Rabbit polyclonal anti-LMW-PTP antibodies were produced in Raugei laboratory, anti-actin antibodies were from Santa Cruz Biotechnology Inc. (Heidelberg Germany), anti-CXCR4 (C-X-C chemokine receptor type 4) polyclonal antibodies were from Sigma-Aldrich (St. Louis, MO, USA), anti-CXCR4 monoclonal antibodies were from Abnova (Aachen, Germany), anti-CD49d-PE antibodies used for VLA-4 detection were from BD. Secondary peroxidase-labeled antibodies were from Santa Cruz Biotechnology. Human CXCL12 (C-X-C motif chemokine 12), fibronectin (FN), fludarabine and morin were purchased from Sigma-Aldrich; ibrutinib from Selleckchem (Munich, Germany).

### Immunoblotting

Cells were lysed on ice in 1× Laemli Buffer (0.5 mol/L Tris–HCl pH 6.8, 10% SDS, 20% glycerol, β-mercaptoethanol, 0.1% bromophenol blue), and samples were boiled for 10 min. Cell extracts were resolved by SDS-PAGE and transferred to PVDF membranes (Bio-Rad Laboratories Segrate Milan, Italy). Membranes were incubated overnight at 4 °C with the appropriate primary antibody. After washing in TPBS-Tween-20 (0.1%), membranes were incubated with the appropriate horseradish peroxidase-conjugated secondary antibodies (Santa Cruz Biotechnology) for 1 h. Proteins were detected using Clarity Western ECL (Bio-Rad) by Amersham 6000 (GE Healthcare).

### Cell transfection

Mec-1 cells (1 × 105 cells/mL) were grown for 24 h and then transiently transfected with LMW-PTP siRNA (target sequence CCCATAGTGCACACTTGTATA), using the Hiperfect Transfection Reagent (Qiagen Italia, Milano, Italy) according to the manufacturer’s instructions. Briefly, the cells were transfected for 24 h with siRNA at a final concentration of 20 nmol/L. To test the specificity of LMW-PTP transfection, control cells were transfected with a scramble sequence (AllStars Negative Control siRNA; at a final concentration of 20 nmol/L; Qiagen) used as negative control. Immunoblotting assessed the efficiency of transfection.

### Apoptosis evaluation

Apoptosis was determined using Annexin-V-FLUOS Staining kit from Roche according to manufacturer’s instructions. 1 × 106 cells were washed in phosphate buffered saline (PBS), and centrifuged at 1300×*g* for 5 min. Cell pellet was resuspended in 100 μL of Annexin-V-FLUOS labeling solution and incubated for 10–15 min at room temperature in the dark. Five hundred microliters of incubation buffer were added and cells were analyzed by flow cytometry using a BDFACS Canto.

### Analysis of receptor recycling, cell adhesion and chemotaxis

Flow cytometry was carried out using a Guava Easy Cyte (Millipore) cytometer. Analysis of CXCR4 was carried out using fluorochrome-conjugated antibodies or isotype control used as negative control on cells fixed and permeabilized using the Cytofix/Cytoperm plus kit (BD). CXCR4 recycling following antibody-dependent downregulation was quantitated by flow cytometry as described [19]. Briefly, cells were incubated for 30 min on ice with CXCR4-specific antibodies, washed, shifted to 37 °C for 40 min, then subjected to acid stripping (time 0), and incubated for the indicated times at 37 °C. Receptor:antibodies complexes that had recycled to the cell surface were measured by labeling with fluorochrome-conjugated secondary antibodies.

Adhesion assays on FN-coated plates in the presence or absence of 100 ng/mL CXCL12 were performed as previously described [20]. Briefly, 48-well plates were coated o/n at 4 °C with 10 mg/mL FN, washed with PBS and incubated for 30 min at 37 °C with RPMI 1% bovine serum albumin (BSA). Then, 2 × 105 cells/well serum-starved Mec-1 cells were added. The plates were incubated at 37 °C for 10 min, then added with 100 ng/mL CXCL12 for further 10 min. Cells that had not adhered (recovered in medium and washes) were resuspended in 0.2 mL RPMI 7.5% BCS. Cells that remained adherent after 3 washes were recovered by 1-min incubation with trypsin/EDTA, immediately added with RPMI 7.5% BCS, washed and resuspended in 0.2 mL RPMI 7.5% BCS. Cells were counted by flow cytometry. The percentage of adherent cells was calculated as previously described [20]. Chemotaxis assays were carried out using 24-well transwell chambers with 5-µm pore polycarbonate membranes (Corning Life Sciences, Schiphol-Rijk, The Netherlands) as described [21]. Briefly, filters were soaked overnight in chemotaxis medium (RPMI 1% BSA). 500 µL of chemotaxis medium with or without 100 nM CXCL12 was placed in the lower chamber, and 100 µL of the cell suspension (5 × 105 cells/sample) in chemotaxis medium was placed in the upper chamber. Samples without chemokine were used as negative controls. After 3 h of incubation at 37 °C in humidified air with 5% CO_2_, the upper chamber was emptied, filters were removed, and the cells in the lower chamber were counted by flow cytometry. The migration index was calculated by determining the ratio of migrated cells in treated versus untreated samples.

### RNA purification and real-time PCR

Total RNA was extracted from Mec-1 cells and retrotranscribed as previously described [22]. Two independent reverse transcription reactions were performed on each RNA sample. Quantitative real-time polymerase chain reaction (qRT-PCR) was performed in triplicate on each cDNA on 96-well optical PCR plates (Sarstedt) using SSo Fast EvaGreenR SuperMix (Bio-Rad) according to the manufacturer’s instructions and a CFX96 Real-Time system (Bio-Rad). After an initial denaturation for 3 min at 95 °C, denaturation in the subsequent 42 cycles was performed for 10 s at 95 °C, followed by 30 s of primer annealing at 60 °C. Results were processed and analyzed using CFX Manager Version 1.5 software (Bio-Rad). Transcript levels were normalized to HPRT1, used as a housekeeping gene. Primers used for amplification are listed in Additional file 1: Table S1.

### Statistical analysis

All statistical analyses were performed with Microcal Origin 8, using Student t test; data, reported as the mean ± SD, were considered significant if p values ≤ 0.05.

## Results

### Morin strongly reduces LMW-PTP expression levels in Mec-1 cells

**Fig. 1:**
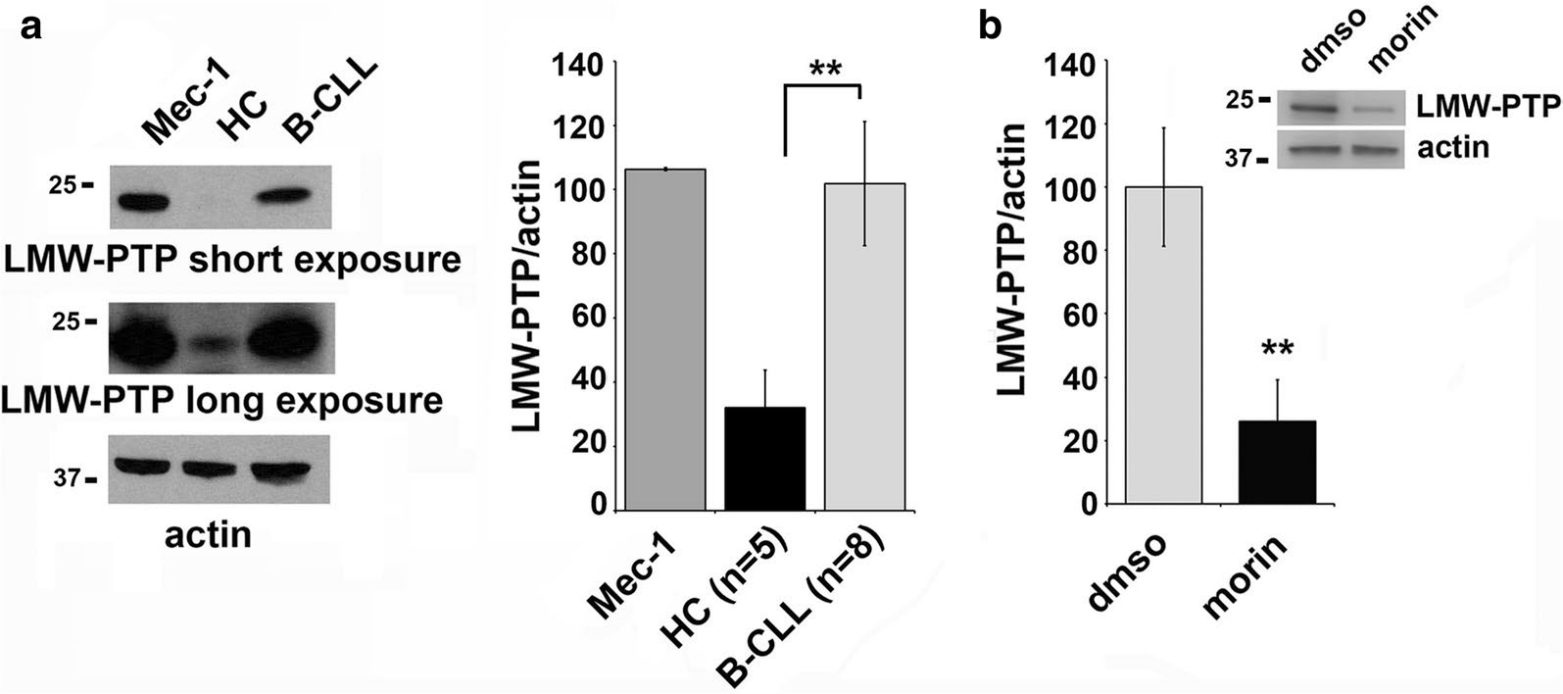
LMW-PTP phosphatase expression is downregulated by morin treatment in Mec-1 cells. **a** Left: Immunoblot analysis with anti-LMW-PTP antibodies from lysates of Mec-1 cells and primary B cells purified from peripheral blood of a representative healthy control (HC) or CLL patient (B-CLL). The stripped filters were reprobed with anti-actin antibodies as loading control. Right: quantification by laser densitometry of the protein bands. Each sample was normalized to the relative actin and data are expressed as percentage (value of Mec-1 cells set as 100). The quantification of protein levels in Mec-1 cells are relative to three independent experiments. For primary B cells the number of samples is HC n = 5 and B-CLL n = 9. Data are expressed as mean ± SD. **b** Quantification by laser densitometry of the LMW-PTP protein levels normalized to the respective actin in Mec-1 cells treated with 50 μM morin or DMSO as control for 24 h. A representative immunoblot analysis is shown on the top of the panel. The quantifications are relative to three independent experiments (n = 3). Error bars, SD. **p ≤ 0.01

LMW-PTP is overexpressed in several solid cancer types and its expression is related to tumour onset and progression [2, 23], but little is known about the LMW-PTP expression levels in human B cells [24]. We quantitated by immunoblot the expression levels of LMW-PTP in a variety of human B cell lines and we observed that LMW-PTP was expressed at high levels in some of them including the CLL-derived Mec-1 cell line (Additional file 1: Figure S1). Moreover, we assessed LMW-PTP protein levels in purified B lymphocytes from peripheral blood of healthy controls (HC) or CLL patients (B-CLL), in comparison with Mec-1 cells. As shown in Fig. 1a, LMW-PTP expression was strongly increased in CLL B cells compared with normal B cells, as well as in the CLL-derived cell line Mec-1, suggesting a relevant role for this phosphatase in CLL pathogenesis (**p ≤ 0.01). Interestingly, 24 h treatment of Mec-1 cells with 50 μM morin resulted in a strong decrease in LMW-PTP expression (Fig. 1b) compared to untreated control, that could be relevant for CLL disease progression and treatment (**p ≤ 0.01). The negative effect of morin on LMW-PTP protein expression is not specific for Mec-1 cells. It was indeed also observed in the lymphoblastoid cell line EBV-B (Additional file 1: Figure S2), which expresses LMW-PTP levels comparable to Mec-1 cells (Additional file 1: Figure S1).

### Morin treatment ameliorates conventional drug-induced apoptosis in Mec-1 leukemic cells by targeting LMW-PTP expression

**Fig. 2:**
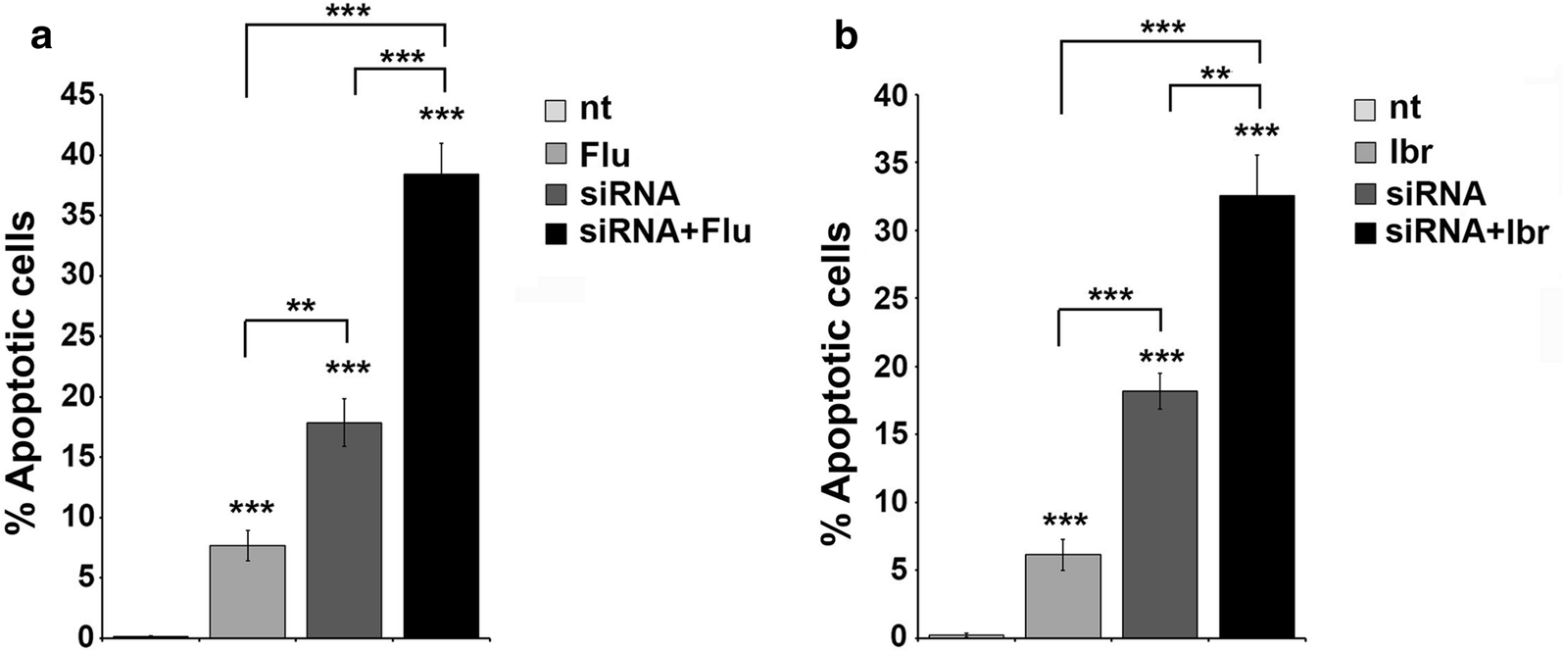
LMW-PTP knockdown results in enhanced Mec-1 cell apoptosis. **a**, **b** Flow cytometric analysis of the percentages of Annexin V^+^/PI^−^ in Mec-1 cell silenced (siRNA) or not for LMW-PTP, treated with either DMSO or 3.5 µM fludarabine (**a**) or 0.1 µM ibrutinib (**b**) for 24 h. Data are expressed as the mean values ± SD determined from three independent experiments. ***p ≤ 0.001, **p ≤ 0.01

Previous reports demonstrated that high levels of LMW-PTP were usually associated to aggressive cancer and induced resistance to chemotherapy, effects that could be strongly counteracted by LMW-PTP silencing [6, 7]. We first assessed the effectiveness of siRNA-mediated LMW-PTP knock-down (Additional file 1: Figure S3). We then assessed the effects of LMW-PTP silencing on Mec-1 cell viability, alone or in combination with fludarabine or ibrutinib, by Annexin V staining. As shown in Fig. 2, LMW-PTP silencing alone was able to strongly induce apoptosis of Mec-1 cells compared to untreated cells (nt) used as negative control, more than that observed when cells were treated with fludarabine (Fig. 2a) or ibrutinib (Fig. 2b) alone. Interestingly, the combination of fludarabine or ibrutinib treatment with LMW-PTP silencing induced significative higher levels of cell death (***p ≤ 0.001, **p ≤ 0.01, *p ≤ 0.05). These data demonstrated that LMW-PTP silencing increases cell death alone and in combination with conventional drugs in Mec-1 cells.

**Fig. 3:**
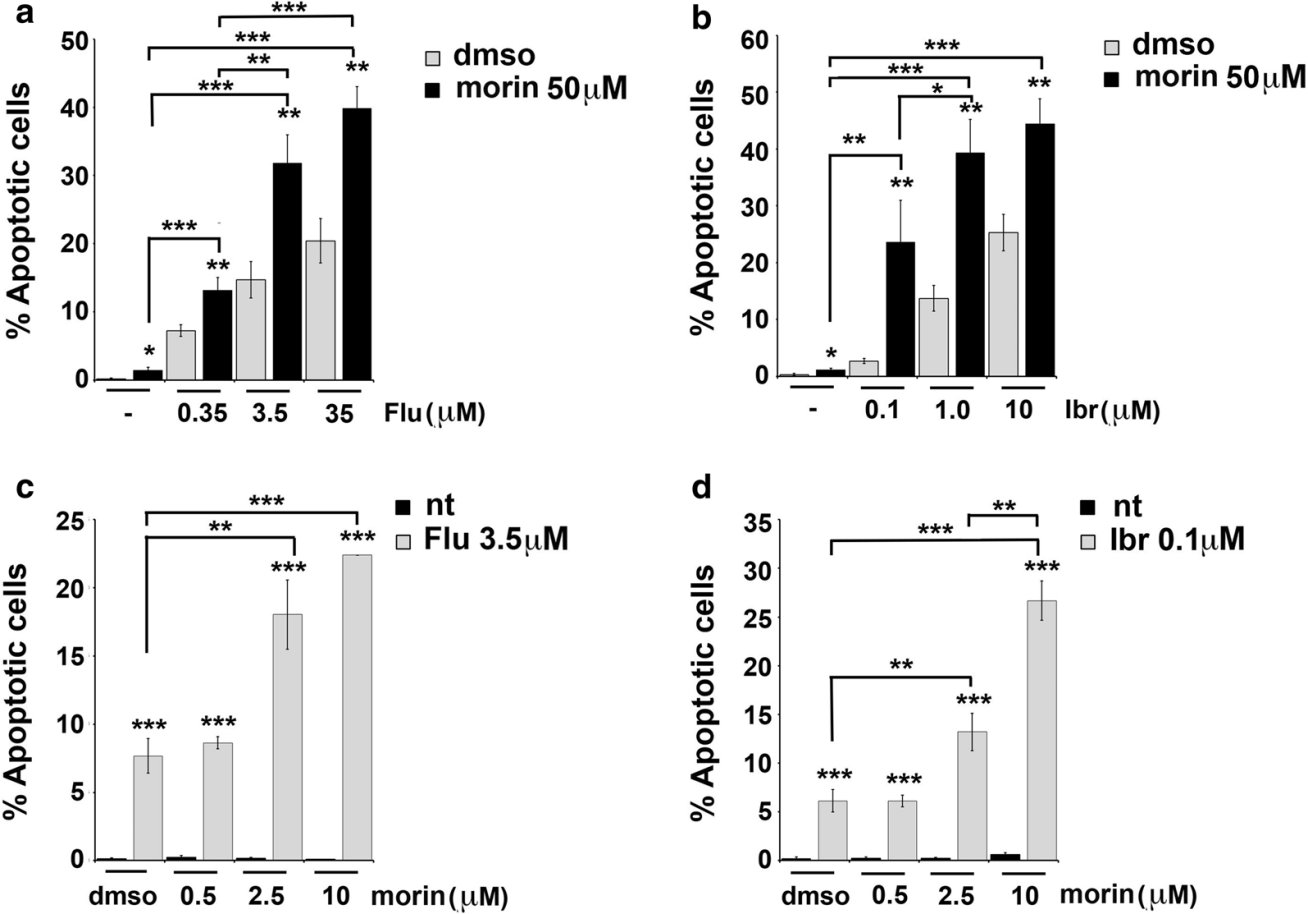
Morin treatment enhances fludarabine- or ibrutinib-induced apoptosis in Mec-1 cells. **a**, **b** Flow cytometric analysis of the percentages of Annexin V^+^/PI^−^ in Mec-1 cell samples treated with either DMSO or 50 µM morin, in the absence or presence of fludarabine (ranging from 0.35 to 35 µM, **a**) or ibrutinib (ranging from 0.1 to 10 µM, **b**) for 24 h. **c**, **d** Flow cytometric analysis of the percentages of Annexin V^+^/PI^−^ in Mec-1 cell samples treated with either DMSO or 3.5 µM fludarabine (**c**) or 0.1 µM ibrutinib (**d**), in the absence or presence of morin (ranging from 0.5 to 10 µM). Data reported in the histogram on the right represent the mean values ± SD determined from three independent experiments. ***p ≤ 0.001, **p ≤ 0.01, *p ≤ 0.05

Because of the fact that morin is known to induce apoptosis in different types of cancer cells [17, 25] and that morin treatment was able to downregulate the expression levels of LMW-PTP in Mec-1 cells (Fig. 1b), we tested the effect of morin alone or in combination with fludarabine or ibrutinib on Mec-1 cell viability. Morin alone did not enhance apoptosis of Mec-1 cells beyond the low basal levels, compared to “dmso” sample used as negative control (Fig. 3a, b). Interestingly, treatment of Mec-1 cells with 50 µM morin in combination with increasing concentrations of fludarabine (ranging from 0.35 to 35 µM, see Fig. 3a) or ibrutinib (ranging from 0.1 to 10 µM, see Fig. 3b) induced a significant increase in cell death compared to fludarabine or ibrutinib alone (***p ≤ 0.001, **p ≤ 0.01). Similar results were obtained treating Mec-1 cells with suboptimal concentrations of fludarabine (3.5 µM) or ibrutinib (0.1 µM) and increasing the concentration of morin, ranging from 0.5 µM to 10 µM (see Fig. 3c, d).

Taken together these data demonstrate that morin, by reducing the LMW-PTP expression, enhances the sensitivity of Mec-1 cells to pro-apoptotic drugs.

Morin amplifies the inhibitory effects of conventional drugs on B-cell adhesion and migration.

The stromal microenvironment has emerged as key player in CLL pathogenesis [26], exerting the double function of creating a proliferative niche for leukemic cells and protecting them from the action of conventional chemotherapeutic drugs. Homing to lymphoid organs is strongly potentiated in CLL B cells, thereby prolonging leukemic cell residency into these protective niches [27]. Integrins and chemokine receptors are both implicated in B-cell homing, by regulating adhesion to endothelial cells and transendothelial migration, respectively [28, 29]. B-cell adhesion is mainly regulated by the integrin Very Late Antigen-4 (VLA-4), which interacts with its ligands vascular cell adhesion molecule 1 (VCAM-1) and FN.

**Fig. 4:**
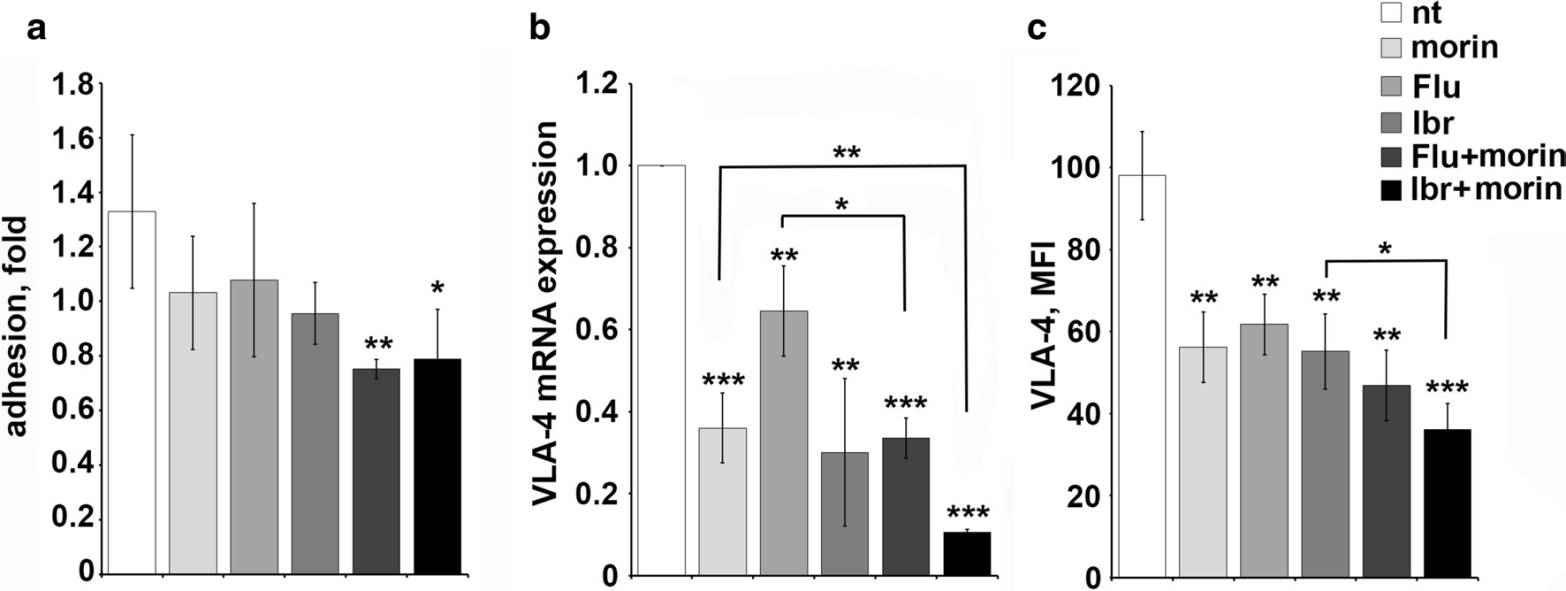
Morin treatment in combination with conventional drugs impairs CXCR4-dependent adhesion and VLA-4 expression in Mec-1 cells. **a** Quantification by flow cytometry of the percentage of Mec-1 cells untreated or treated with 0.1 μM ibrutinib or 3.5 μM fludarabine or 50 μM morin or the combination of morin with either fludarabine or ibrutinib for 24 h, that adhered to 48-well plates coated with 10 μg/mL fibronectin following a 10 min ìtreatment with 100 ng/mL CXCL12. The data, which refer to quadruplicate samples from three independent experiments, are presented as fold of adherent cells in CXCL12 stimulated samples versus the corresponding unstimulated controls. **b** Quantitative RT-PCR analysis of VLA-4 mRNA on Mec-1 cells treated as in panel A. The relative gene transcript abundance was determined on triplicate samples using the ddCt method (n = 3). **c** Flow cytometric analysis of VLA-4 on Mec-1 cells treated as in panel A and permeabilized (n = 3). Error bars, SD; ***p ≤ 0.001, **p ≤ 0.01, *p ≤ 0.05

The effect of morin on leukemic cell adhesion was addressed treating Mec-1 cells with 0.1 μM ibrutinib or 3.5 μM fludarabine for 16 h and then adding or not 50 μM morin for 6 h at 37 °C. Cells were subsequently plated on immobilized FN in the presence or absence of CXCL12 and the proportion of cells that had adhered after a short incubation was determined by flow cytometry. Morin treatment alone negatively regulated CXCR4-dependent adhesion to FN compared to the untreated sample (nt) used as negative control, with an effect comparable to the treatment with the conventional drugs fludarabine and ibrutinib (Fig. 4a). Interestingly the combination of morin with either fludarabine or ibrutinib was more effective at inhibiting the ability of Mec-1 cells to adhere to FN compared with single agent treatments. Consistent with this finding, real time and cytofluorometric analysis for VLA-4 expression revealed a strong reduction of transcript and protein levels in Mec-1 cells treated with 0.1 μM ibrutinib or 3.5 μM fludarabine or 50 μM morin or the combination of morin with either fludarabine or ibrutinib for 24 h (Fig. 4b, c). Statistical significance is indicated (***p ≤ 0.001, **p ≤ 0.01, *p ≤ 0.05).

**Fig. 5:**
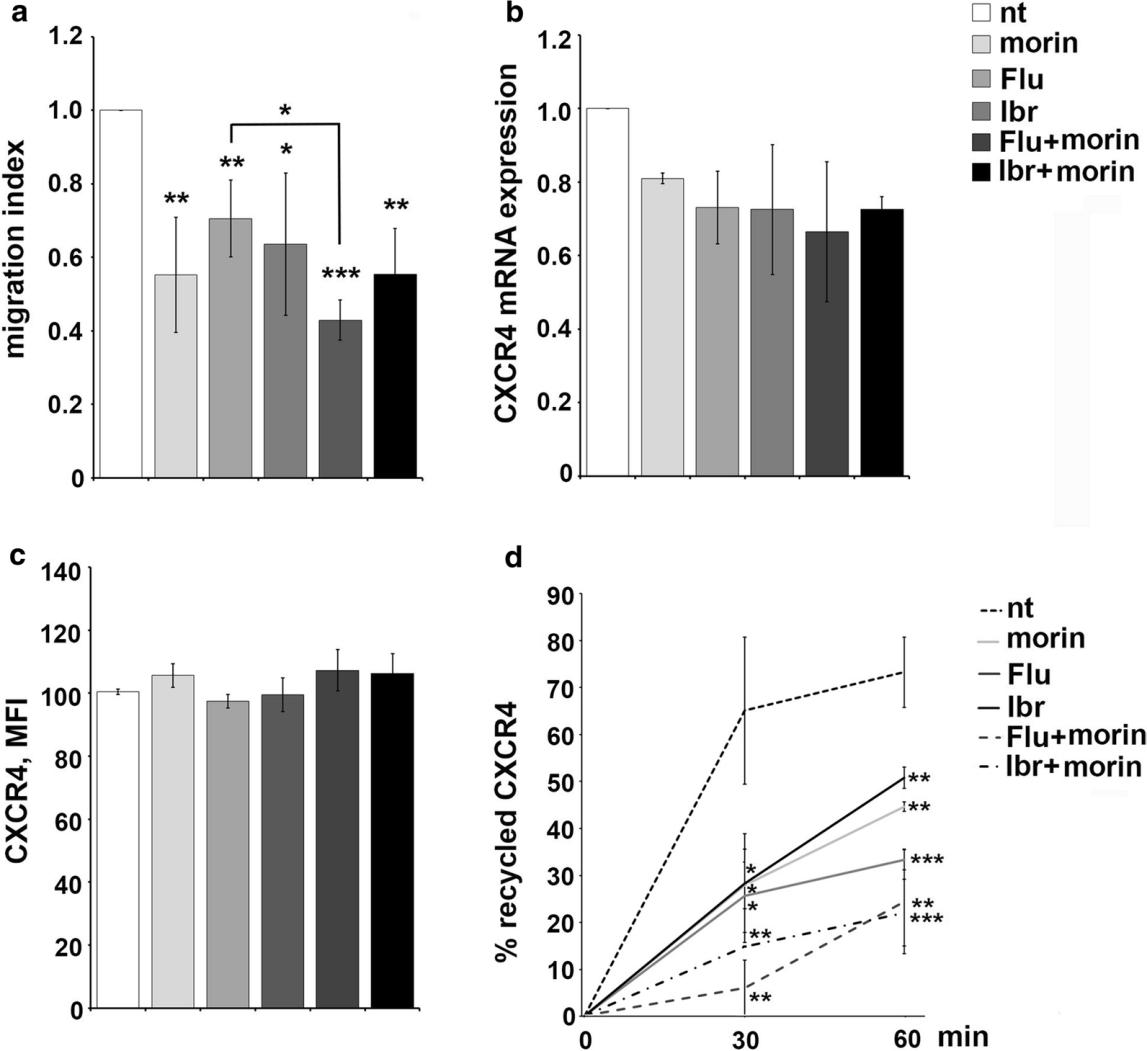
Morin treatment in combination with conventional drugs inhibit CXCR4-dependent migration and CXCR4 receptor recycling in Mec-1 cells. **a** Migration of Mec-1 cells treated as in Fig. 4, stimulated or not for 3 h with 100 ng/mL CXCL12. The data, obtained on duplicate samples from at least three independent experiments, are presented as mean migration index ± SD (ratio of migrated cells in chemokine-treated versus untreated samples). **b** Quantitative RT-PCR analysis of CXCR4 mRNA on Mec-1 cells treated as in Fig. 4. The relative gene transcript abundance was determined on triplicate samples using the ddCt method (n = 3). **c** Flow cytometric analysis of CXCR4 on Mec-1 cells treated as in Fig. 4 and permeabilized (n = 3). **d** Flow cytometric analysis of CXCR4 recycling in Mec-1 cells treated as in Fig. 4. Data are presented as  % of internalized receptors that have recycled to the cell surface and refer to duplicate samples (n = 3). Error bars, SD; ***p ≤ 0.001, **p ≤ 0.01, *p ≤ 0.05

The effect of morin treatment on B-cell chemotaxis was addressed in transwell migration assays, using CXCL12 as chemoattractants. CXCR4-dependent migration was impaired in morin treated Mec-1 cells compared with untreated cells used as negative control, as well as in Mec-1 cells treated with 0.1 μM ibrutinib or 3.5 μM fludarabine as single agents (Fig. 5a). The migratory potential was further inhibited when cells were treated with the combination of morin and fludarabine or ibrutinib (Fig. 5a). qRT-PCR and cytofluorometric analysis of CXCR4 expression in Mec-1 cells did not reveal any significant modulation in the transcript or protein levels following morin treatment alone or in combination with fludarabine or ibrutinib (Fig. 5b, c). The relative gene transcript abundance was determined using the ddCt method, relative to the untreated sample (nt). Statistical significance is indicated for panel a and d (***p ≤ 0.001, **p ≤ 0.01, *p ≤ 0.05).

CXCR4 expression at the plasma membrane is dynamically regulated by its downmodulation in the presence of high ligand concentrations and its recycling when these decrease [30], and it was previously demonstrated that enhanced receptor recycling contributes to the increased surface CXCR4 levels on CLL cells and enhanced migratory responses [31]. Interestingly, here we showed that morin treatment strongly inhibited CXCR4 receptor recycling in Mec-1 cells, both as single agent or in combination with fludarabine or ibrutinib (Fig. 5d), accounting for the inhibitory effect observed on CXCR4-dependent migration (Fig. 5a). Hence, morin acts, alone or in combination with fludarabine or ibrutinib, as a negative regulator of both cell adhesion and CXCR4-dependent migration in Mec-1 cells by affecting VLA-4 expression levels and inhibiting CXCR4 receptor recycling, respectively.

## Discussion

LMW-PTP is overexpressed in different cancer types and its expression is related to a worse prognosis and reduced survival rate [2]. Chiarugi et al. [23] showed that LMW-PTP increases engrafted tumor growth in nude mice, confirming a possible role of this enzyme in tumorigenesis also in in vivo models. Moreover independent studies demonstrated that over-expression of LMW-PTP confers resistance to vincristine in leukemic cells [5] and enhances the malignant potential of colorectal cancer cells, inducing drug resistance and modulating cell motility [6].

Here we demonstrated that LMW-PTP phosphatase is expressed at high levels in the CLL-derived Mec-1 B cell line and is overexpressed in primary B cells purified from peripheral blood of CLL cells compared with healthy controls (Fig. 1a).

Because of CLL is primarily a disease of defective apoptosis rather than uncontrolled proliferation [4, 8, 32], introducing in the treatment of this disease agents able to induce leukemic cell death it is one of the main goal that have to be achieved. Here we use the flavonoid morin that was largely demonstrated to be able to sensitize cancer cells to apoptosis, downregulating expression of the main anti-apoptotic proteins and modulating the expression of many enzymes. We found that morin is able to downregulate LMW-PTP levels in Mec-1 cells (Fig. 1b) and that treatment with morin is able to increase Mec-1 cell sensitivity to apoptosis induced by fludarabine or ibrutinib, two drugs currently used in CLL treatment (Fig. 3). The mechanism by which morin contributes to apoptosis induction appears to be related to its ability to reduce LMW-PTP levels, since LMW-PTP knock down itself results in enhanced leukemic cell death (Fig. 2).

The stromal microenvironment has emerged as a key player in CLL cell survival and expansion [32–34]. B lymphocyte homing to SLOs and BM is regulated by a balance between homing receptors, such as CXCR4 and CCR7 [35, 36], and receptors that promote lymphocyte egress from their homing sites, such as sphingosine-1-phosphate receptor 1 (S1PR1) [37]. CLL cells display an altered balance in the surface expression of these receptors toward the homing ones [31, 38, 39], resulting in the prolonged residency of leukemic cell in the pro-survival stromal niche of SLOs where they are subjected to survival signal and protected from chemotherapy [40]. Hence, searching for compounds that are able to restore normal lymphocyte trafficking by inhibiting the enhanced adhesion and migration observed in leukemic cells remains one of the principal tasks in CLL treatment. Ibrutinib alone was previously demonstrated to have a role in CLL cell trafficking by regulating the expression levels of homing receptors [39] and in the regulation of CXCR4 receptor recycling [26]. Here we observed that morin was able to synergize with fludarabine and ibrutinib in decreasing the ability of Mec-1 cells to adhere to FN (Fig. 4a) and to migrate toward a CXCL12 gradient (Fig. 5a), by regulating respectively VLA-4 integrin expression levels (Fig. 4b) and CXCR4 recycling (Fig. 5d).

Further experiments are required to assess whether LMW-PTP over-expression correlates with a faster disease progression and/or resistance to pharmacological treatments, and if it may be predictive for the overall survival. In addition, in order to explore the potential exploitation of Morin as adjuvant therapy to small molecule drugs and/or biologicals currently used for CLL treatment, it will be mandatory to carry out in vivo experiments using appropriate animal models of CLL, such as the Eμ-TCL1 mouse [41, 42].

## Conclusions

These data provide a rationale for using morin as sensitizing agent to improve the effectiveness of traditional therapies by inducing apoptosis of leukemic cells and by modulating their ability to recirculate from SLOs to peripheral blood where they are more susceptible to conventional anticancer drugs.

## Additional file

**Additional file 1: Table S1.** List of the primers used in this study. **Figure S1.** *Left.* Immunoblot analysis with anti-LMW-PTP antibodies from lysates of a variety of B cell lines. The stripped filters were reprobed with anti-actin antibodies as loading control. *Right.* Quantification by laser densitometry of the protein bands. Each sample was normalized to the respective actin and data are expressed as percentage (value of Mec-1 cells set as 100). Data are expressed as mean ± SD. **Figure S2.** Quantification by laser densitometry of the LMW-PTP protein levels normalized to the respective actin in EBV-B cells treated with 50 μM morin or DMSO as control for 24 h. A representative immunoblot analysis is shown on the top of the panel. The quantifications are relative to three independent experiments. Error bars, SD. ***p ≤ 0.001. **Figure S3.** Quantification by laser densitometry of the LMW-PTP protein levels normalized to the respective actin in Mec-1 cells transfected with LMW-PTP siRNA or scramble. A representative immunoblot analysis is shown on the top of the panel. The quantification are relative to three independent experiments. Error bars, SD. ***p ≤ 0.001.

## Abbreviations

LMW-PTP: low molecular weight protein tyrosine phosphatase
siRNA: small interference RNA
CLL: chronic lymphocytic leukemia
EBVB: Epstein–Barr virus B cells
RPMI: Roswell Park Memorial Institute
BCS: bovine calf serum
CXCR4: C-X-C chemokine receptor type 4
CXCL12: C-X-C motif chemokine 12
FN: fibronectin
PBS: phosphate buffered saline
BSA: bovine serum albumin
qRT-PCR: quantitative real-time polymerase chain reaction
Btk: bruton tyrosine kinase
VLA-4: very late antigen-4
VCAM-1: vascular cell adhesion molecule 1
