# Supplementary material for: LMW-PTP targeting potentiates the effects of drugs used in chronic lymphocytic leukemia therapy
Source: Cancer Cell Int. 2019 Mar 21;19:67. doi: 10.1186/s12935-019-0786-1 (PMC6429822; doi:10.1186/s12935-019-0786-1)
Supplement: Supplementary file 1 — Additional file 1: Table S1. List of the primers used in this study. Figure S1. Left. Immunoblot analysis with anti-LMW-PTP antibodies from lysates of a variety of B cell lines. The stripped filters were reprobed with anti-actin antibodies as loading control. Right. Quantification by laser densitometry of the protein bands. Each sample was normalized to the respective actin and data are expressed as percentage (value of Mec-1 cells set as 100). Data are expressed as mean ± SD. Figure S2. Quantification by laser densitometry of the LMW-PTP protein levels normalized to the respective actin in EBV-B cells treated with 50 μM morin or DMSO as control for 24 h. A representative immunoblot analysis is shown on the top of the panel. The quantifications are relative to three independent experiments. Error bars, SD. ***p ≤ 0.001. Figure S3. Quantification by laser densitometry of the LMW-PTP protein levels normalized to the respective actin in Mec-1 cells transfected with LMW-PTP siRNA or scramble. A representative immunoblot analysis is shown on the top of the panel. The quantification are relative to three independent experiments. Error bars, SD. ***p ≤ 0.001. [file 12935_2019_786_MOESM1_ESM.docx]

**Table S1. List of the primers used in this study**

| **Quantitative RT-PCR** | **Forward 5’-3’** | **Reverse 5’-3’** |
| --- | --- | --- |
| VLA-4 | CTG CTG TTG CTG CTG CTG | TCC ACT GAG AAT CCG AAG AAG G |
| CXCR4 | TGG TCT ATG TTG GCG TCT GG | GAA CAC AAC CAC CCA CAA GTC |
| HPRT1 | AGA TGG TCA AGG TCG AAG | GTA TTC ATT ATA GTC AAG GGC ATA TC |

**Figure S1.** *Left.* Immunoblot analysis with anti-LMW-PTP antibodies from lysates of a variety of B cell lines. The stripped filters were reprobed with anti-actin antibodies as loading control. *Right.* Quantification by laser densitometry of the protein bands. Each sample was normalized to the respective actin and data are expressed as percentage (value of Mec-1 cells set as 100). Data are expressed as mean ± SD.

**Figure S2.** Quantification by laser densitometry of the LMW-PTP protein levels normalized to the respective actin in EBV-B cells treated with 50 μM morin or DMSO as control for 24 h. A representative immunoblot analysis is shown on the top of the panel. The quantifications are relative to three independent experiments. Error bars, SD. *** p≤0.001.

**Figure S3.** Quantification by laser densitometry of the LMW-PTP protein levels normalized to the respective actin in Mec-1 cells transfected with LMW-PTP siRNA or scramble. A representative immunoblot analysis is shown on the top of the panel. The quantification are relative to three independent experiments. Error bars, SD. *** p≤0.001.


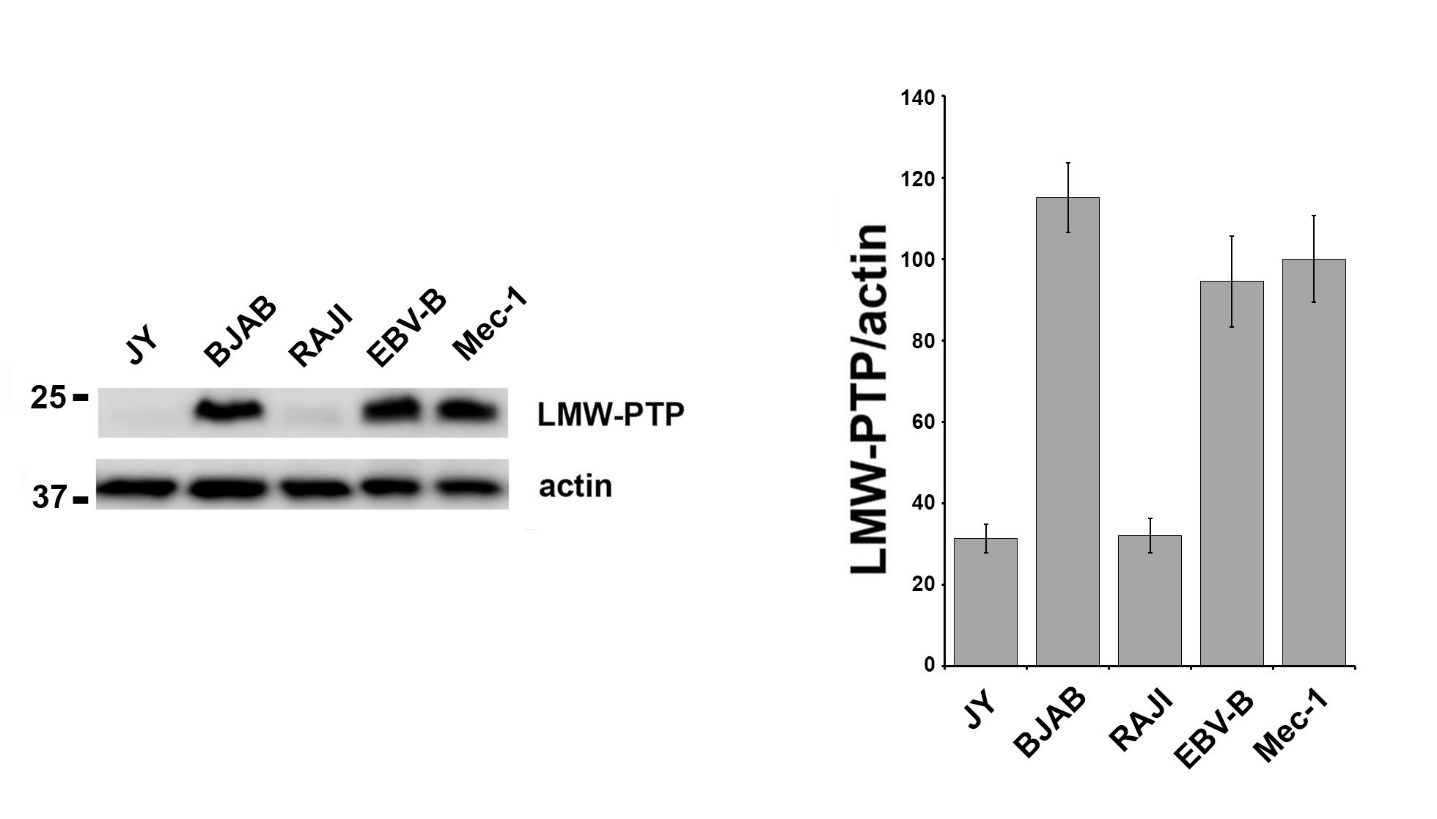


Figure S1


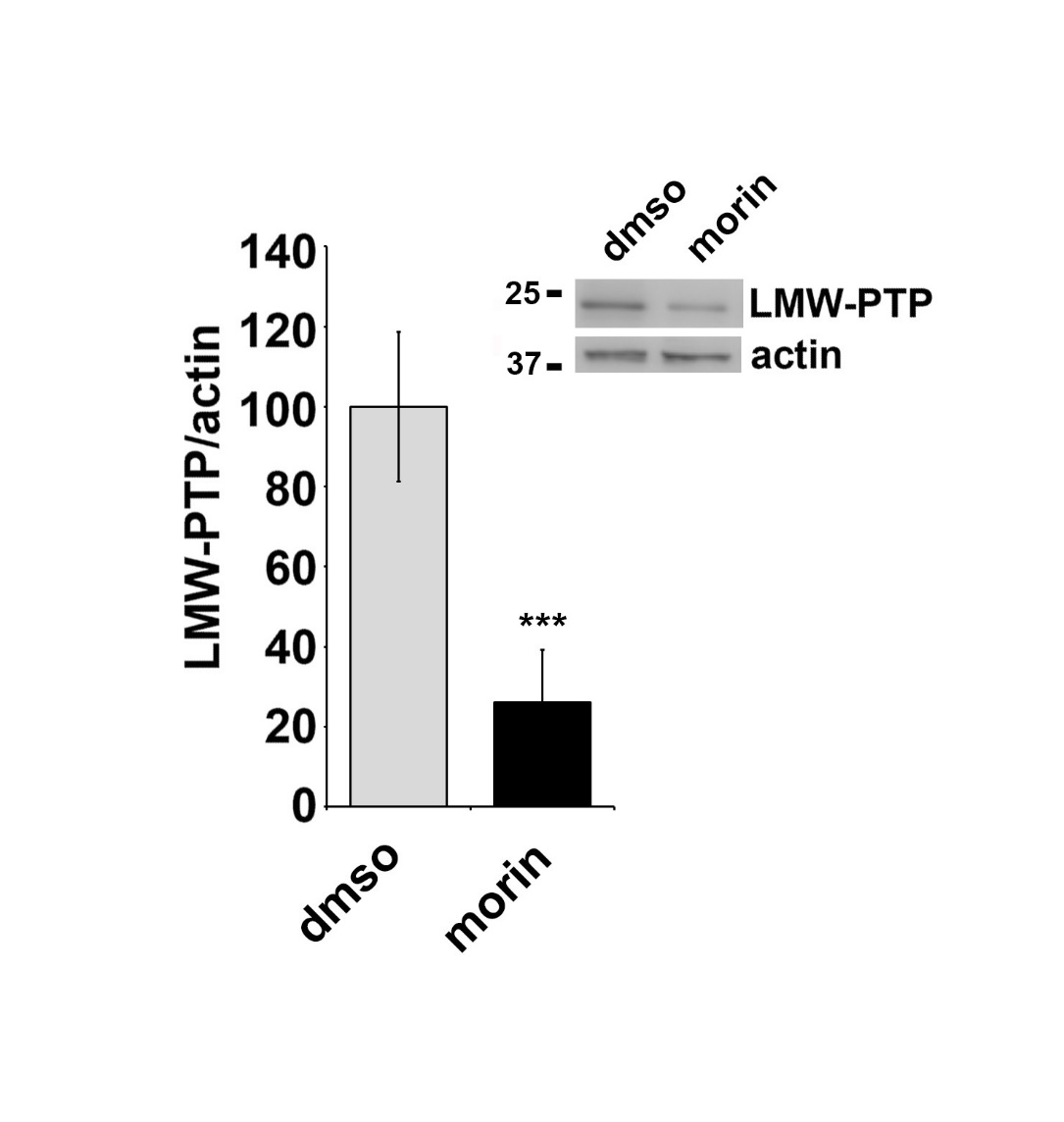


Figure S2


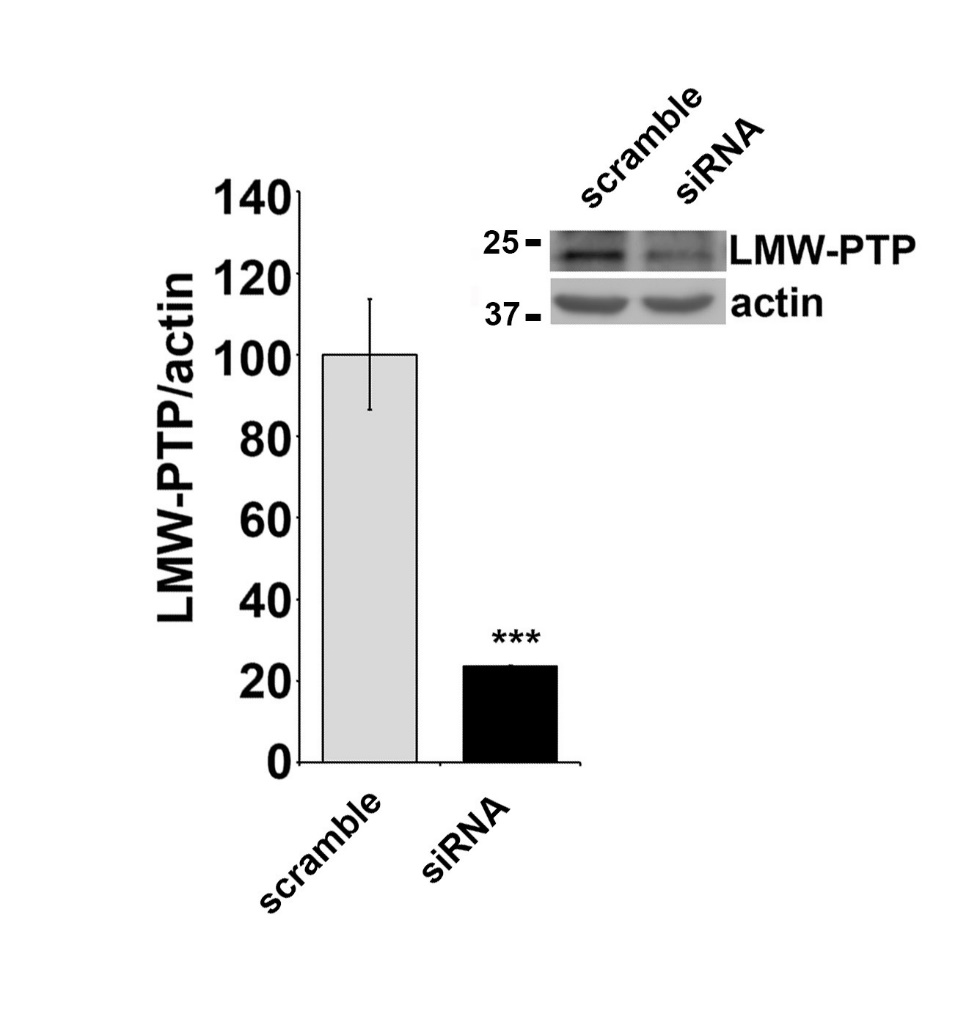


Figure S3
